# Climate of Hate: Similar Correlates of Far Right Electoral Support and Right-Wing Hate Crimes in Germany

**DOI:** 10.3389/fpsyg.2019.02328

**Published:** 2019-10-18

**Authors:** Jonas H. Rees, Yann P. M. Rees, Jens H. Hellmann, Andreas Zick

**Affiliations:** ^1^Institute for Interdisciplinary Research on Conflict and Violence, Bielefeld University, Bielefeld, Germany; ^2^Department of Social Psychology, Bielefeld University, Bielefeld, Germany; ^3^Department of Psychology, Westfälische Wilhelms-Universität Münster, Münster, Germany

**Keywords:** right-wing extremism, populist parties, intergroup contact, collective deprivation, socio-structural variables

## Abstract

Since 2015, far right parties drawing heavily on radical anti-refugee rhetoric gained electoral support in Germany while the number of political hate crimes targeting refugees rose. Both phenomena – far right electoral support and prevalence of right-wing hate crimes – have theoretically and empirically been linked with socio-structural and contextual variables. However, systematic empirical research on these links is scattered and scarce at best. We combine official statistics on political hate crimes targeting refugees in Germany and far right electoral support of the far right party “Alternative für Deutschland” (AfD) in the German national elections 2017 with socio-structural variables (proportion of foreigners and unemployment rate) and survey data collected in a representative survey (*N* = 1,506) in 2016. We aggregate and combine data for all German municipalities except Berlin which were the level of analysis for the current study. In path analyses, we find socio-structural variables to be unrelated with each other but significantly correlated with both criterion variables in a systematic fashion: proportion of foreigners was negatively while unemployment rate was positively linked with far right electoral support. Right-wing crime was linked positively with unemployment rate across Germany and positively with proportion of foreigners only in East Germany while proportion of foreigners was unrelated to right-wing crime in West Germany. When including survey measures into the model, they were linked with socio-structural variables in the predicted fashion – intergroup contact correlated positively with proportion of foreigners, collective deprivation correlated positively with unemployment rates, and both predicted extreme right-wing attitudes. However, their contribution to the explained variance in outcome variables above and beyond socio-structural variables was neglectable. We argue that both far right-wing electoral support and right-wing hate crime can be conceptualized as behavioral forms of political extremism shaped through socio-structural and contextual factors and discuss implications for preventing political extremism.

“The one thing […] that is truly ugly is the climate of hate and intimidation, created by a noisy few, which makes the decent majority reluctant to air in public their views on anything controversial.”

– Edward Abbey

## Introduction

Much has been written about the recent wave of success for far right, right-wing populist and extreme right-wing parties, figures and movements globally but especially in the Western world. There seems to be agreement that we are witnessing what some scholars have called a “revolt against liberal democracy” (Eatwell and Goodwin, 2018), a “cultural backlash” (Norris and Inglehart, 2019), or – in more technical and less alarming words – growing support for the far right (Golder, 2016). These developments seem to temporally coincide with an increase in hate-crime in countries across the world (see, e.g., Osce Hate Crime Reporting, 2019) and a number of right-wing terrorist attacks that have gained media attention around the world: Attacks in Oslo and Utøya, 2011, Charleston, 2015, and Christchurch, 2019 among others have been directly linked with extreme right-wing ideology. While there is by now a rich literature on far right electoral support, its potential links with right-wing crimes are not well understood. In public discourse and discussions amongst practitioners, there seems to be an implicit assumption that both phenomena – far right electoral support and prevalence of right-wing hate crimes – are related (e.g., Chu, 2018). However, systematic empirical research on such potential links is scattered and scarce at best. In the current paper, we argue that both are not independent phenomena but have similar correlates and are also correlated with each other. We combine official statistics on reported right-wing hate crimes targeting refugees in Germany and far right electoral support in the German national elections 2017 and investigate links with socio-structural variables (proportion of foreigners and unemployment rate) on the one hand and with psychological variables measured in a representative survey (perceived threat, intergroup contact, and extreme right-wing attitudes) on the other hand.^1^ We show that both phenomena co-occur geographically, and we do so in the German context where right-wing hate crime has recently peaked while the far right has seen increasing electoral support in the aftermath of the immense refugee in-migration since 2015.

### The German Context

In recent years, wars and other conflicts including personal persecution, primarily in the Middle East and Africa have forced millions of individuals to leave their original places of residence. While most refugees temporarily settle in neighboring countries like Lebanon or Turkey, many have also migrated to Western Europe. Almost 1 million refugees have sought asylum in Germany in the year 2015 alone (German Federal Office for Migration and Refugees, 2019). Germany was not only the European country in which most refugees applied for asylum (Eurostat, 2017). It has also been at the center of attention for a number of events during this period, most prominently for its “welcome culture”. As one example, German chancellor Angela Merkel famously announced that several thousand refugees would be allowed to cross the border from Hungary to Austria and into Germany in September 2015 (e.g., Hall and Lichfield, 2015). Her public press announcement “Wir schaffen das!” (We can do this) became historical. While solidarity and the willingness to help have generally been high (e.g., Akrap, 2015), Germany also experienced a wave of hostile and violent resistance against refugees (e.g., Benček and Strasheim, 2016). The number of political hate crimes targeting refugees and their homes in Germany rose dramatically and peaked in 2016 with a total of more than 3,000 incidents according to official sources with an unclear number of incidents that remained in the dark field (Federal Government, 2017; ProAsyl, 2017).^2^ Such incidents range from propaganda crimes like libel, incitement of the masses and harassment to violent hate crimes like assault, arson and right-wing extremist terrorist attacks.

During the same period, far right parties drawing on radical anti-refugee rhetoric gained electoral support in Germany – most notably the AfD (Alternative für Deutschland, *Alternative for Germany*).

We begin by tracing the rise of this most prominent current far right party in Germany, the AfD, and summarize previous research and historical links between socio-structural variables such as unemployment and far right electoral support. We then briefly review two prominent social-psychological factors – perceived threat and intergroup contact – that can help understand both outcomes that are of interest for the current work. As we shall see, theoretically as well as empirically, far right electoral support and right-wing hate crimes have similar socio-structural as well as psychological correlates that can produce a dangerous climate of hate.

### Rise of the AfD

The AfD was founded in 2013. During the first few years after its formation, the party strongly opposed European integration, the European currency “Euro” and especially European assistance programs during the European sovereign-debt crisis (Arzheimer, 2015), such as the *European Stability Mechanism* and the *European Financial Stability Facility*. The party’s “Euro-skeptic” positions broadened during the so called “European migration crisis” from 2015 and onward. Since then, a shift in AfD policy has been reliably documented: The party’s positions drastically changed from an economic critique of the European Union toward a far right ideological, nationalist and anti-immigration course (e.g., Franz et al., 2018). During this period of change, AfD personnel and party leadership also changed. This shift seems to have facilitated electoral success for the AfD. Since 2015, the AfD gained more and more electoral support and was able to consolidate as a relevant political force with a breakthrough in 2016: In the Eastern German states of Saxony-Anhalt (24.2%) and Mecklenburg-Vorpommern (20.8%), the AfD had their biggest electoral successes. However, the AfD also had substantial impact in the in 2016 Western German state elections in Rhineland-Palatinate (12.6%), Berlin (14.2%) and Baden-Wurttemberg (15.1%). This trend culminated in a striking 12.6% in the German general elections in September of 2017 (21.8% in the five states of the former *German Democratic Republic*) establishing the AfD as the third most powerful party and opposition leader in the German Parliament, the *Bundestag*. With the AfD in the Bundestag, the party’s positions and views have entrenched large parts of German society seemingly independent of financial and societal status (Zick et al., 2016; Bergmann et al., 2017; Franz et al., 2018; Schröder, 2018) and support of AfD positions in the German society increased. Various polls currently have the AfD (13–14%) close to the Social Democrats (12%), making the AfD the third most successful party in Germany as of August 2019 (Wahlrecht.de, 2019). Before discussing psychological factors that should be relevant for the AfD’s electoral success as well as right-wing hate crime, we shall now review socio-structural variables that have historically and empirically been linked with far right electoral support.

### Unemployment and Far Right Electoral Support

The link between unemployment and far right electoral support is well-established in the literature and has been empirically demonstrated numerous times. In one of the earliest studies, Pratt (1948) analyzed the 1932 German Reichstag Elections and found that unemployed citizens tended to vote for extreme political parties, such as the Nazi-Party (*NSDAP*), but also the Communist-Party (*KPD*). Falter and Zintl (1988) further corroborated these findings arguing that high unemployment rates facilitated the electoral and overall success of the Nazis in Germany in the 1930s. O’Loughlin et al. (1994), however, showed that these findings seem to hold only for the 1932 and 1933 German general elections but not for the 1930 election. The authors stress the importance of “regional and local contexts of the voting decisions” (O’Loughlin et al., 1994, p. 373). In more recent studies, the general assertion that unemployment and far right electoral support are correlated still holds true (e.g., Norris, 2005; Mudde, 2007; Rydgren, 2007). In his work on far right electoral support, Rydgren (2009) investigated the broader concept of social isolation. Among other factors such as lack of social relations, weak family structures and less personal involvement in civil society, he lists unemployment as a key variable for supporting far right parties (Rydgren, 2009). On the basis of a geographically weighted regression (GWR) analysis focusing on the electoral results of the German Neo-Nazi party NPD (Nationaldemokratische Partei Deutschland), Teney (2012) found a connection between far right electoral outcomes and local unemployment rates while also emphasizing the importance of socio-spatial as well as regional variations among municipalities (Teney, 2012). However, there have been some studies showing that high levels of unemployment do not necessarily have a determining impact on far right voting. Based on election studies, far right voting outcome in seven European countries as well as supranational surveys, for example, Arzheimer and Carter (2006) found that higher unemployment rates are linked with less far right voting. Similarly, Oesch (2008) emphasizes that unemployment is no major factor influencing far right voting, but rather matters of identity claiming that “questions of identity are more important than economic questions” (Oesch, 2008, p. 370). It seems then that while unemployment has been linked with far right electoral support numerous times, it does not tell the full story. In social-psychological theorizing there are two prominent constructs that should be of interest for the current research, that is, intergroup contact and perceived threat.

### Intergroup Contact

According to intergroup contact theory, which was first developed by Allport (1954), opportunities for random encounters or cross-group friendships (e.g., Pettigrew, 1998) reduce negative attitudes and prejudice toward outgroups. Allport originally assumed that certain “optimal conditions” (i.e., equal status, perception of common goals, institutional support, perception of common humanity) would facilitate the positive effects of intergroup contact (Allport, 1954). In a meta-analysis of more than 500 studies, Pettigrew and Tropp (2006) found empirical support for the theory and showed that optimal contact conditions may yield greater reduction in negative attitudes but might not always be necessary to reduce prejudice. Several adaptations and extensions for Allport’s original theory have been suggested (e.g., Wright et al., 1997; Pettigrew, 2009) but there seems to be general agreement that intergroup contact opportunities tend to decrease hostile and negative attitudes toward outgroups in general even in the absence of optimal conditions (but see Barlow et al., 2012).

The proportion of foreigners in a given spatial unit (e.g., municipality) can be considered the most straightforward socio-structural indicator for contact opportunities and varies considerably across Germany (e.g., Wagner et al., 2003, 2006, 2008). For example, the rate of foreigners is still as much as four times lower in Eastern versus Western German federal states (Statistisches Bundesamt, 2018). Such preconditions provide only relatively few opportunities for intergroup contact for Germans in East as compared to West Germany. Contact theory has consequently been widely used as social-psychological theoretical framework explaining higher levels of prejudice (e.g., Decker et al., 2016; Zick et al., 2019) as well as higher rates of xenophobic attacks and hate crimes against foreigners (e.g., Benček and Strasheim, 2016) in East versus West Germany (see also Wagner et al., 2003; Andresen et al., 2018). Furthermore, intergroup contact with specifically refugees is more prevalent in West than in East Germany as revealed in recent large-scale surveys (Ahrens, 2017). Based on intergroup contact theory, one would therefore predict negative links between proportion of foreigners and prejudice as well as far right electoral support (Teney, 2012) and political hate-crime (Benček and Strasheim, 2016). It seems noteworthy that at least this second prediction is somewhat contradictory to the intuition that for hate-crimes to occur, the target outgroup needs to be present. However, according to intergroup contact theory, hate-crimes should be most frequent in areas with low rates of outgroup individuals, that is, for example, foreigners and refugees. In a nutshell, we thus assumed a negative relationship between number of refugees in a particular German municipality and numbers of hate-crimes against refugees. Other theoretical approach that is relevant for the present study concern perceived threat ostensibly posed by the outgroup and their members and collective deprivation.

### Perceived Threat and Collective Deprivation

A major psychological driver of antagonistic intergroup attitudes is the perception that the outgroup threatens the ingroup’s status or culture (e.g., Semyonov et al., 2004; but see Wagner et al., 2006). Perceived threat has consequently also been used to explain differences in prejudice levels between East and West Germans (Wagner et al., 2003; Asbrock et al., 2014; also see Semyonov et al., 2004; Wagner et al., 2008).

Interestingly, threat can be closely related to the socio-structural factor discussed above: Higher unemployment rates in Eastern versus Western German federal states may contribute to differences in perceived threat or status. In other words, competition over jobs and economic opportunities might translate into higher perceived threat by outgroups in general. This perception may, in turn, be linked with prejudice and other negative attitudes toward members of ethnic out-groups. Perceived threat does not necessarily coincide with realistic threat. In response to the recent so-called “refugee crisis”, concerns about immigration increased twice as much in East as compared to West Germany (Sola, 2018; also see Jacobsen et al., 2017). A concept that might therefore be psychologically more relevant in this context is collective or fraternal deprivation (Runciman, 1966; also see Major, 1994; Pettigrew and Meertens, 1995). In his original conceptualization, Runciman (1966) distinguished fraternal from egoistic deprivation and argued that it is linked with “lateral solidarity” or ingroup identification for social groups that are relatively deprived in some objective way – such as areas with higher unemployment. Even more importantly, collective deprivation also “uniquely generates agitation for or against structural change” (Taylor, 2002, p. 15). It should therefore be particularly relevant when it comes to voting for a party that insistently opposes structural and societal change – such as the AfD.

### The Current Study

The aim of the current study was to investigate socio-structural and psychological correlates of far right electoral support and hate crimes in Germany. Due to its administrative organization into 401 municipalities (294 “Landkreise/Kreise” and 107 “kreisfreie Städte”), Germany lends itself to analyses combining data from different sources that are available on this level. We therefore combine socio-structural data that are made available on a regular basis (i.e., unemployment rates and proportion of foreigners per municipality) with other data from official sources (i.e., election results and reported hate crimes targeting refugees and their homes). We also included survey data on intergroup contact, fraternal deprivation, and extreme right-wing attitudes that were collected as part of a representative telephone survey and that could be located on the municipality level.

As a first set of hypotheses, we predicted substantial links between socio-structural factors and both outcome variables. More specifically, based on previous research and theorizing, we expected (Hypothesis 1a) unemployment rate to be positively linked with far right electoral support (e.g., Pratt, 1948; Falter and Zintl, 1988; Jackman and Volpert, 1996; Rydgren, 2009) and (Hypothesis 1b) proportion of foreigners to be negatively linked with right-wing hate crime (e.g., Wagner et al., 2003; Bustikova, 2014; Benček and Strasheim, 2016; Andresen et al., 2018). As the evidence for a link between proportion of foreigners and far right electoral support is mixed (Arzheimer and Carter, 2006; Lubbers et al., 2006; Golder, 2016), we made no predictions regarding this link or the link between unemployment rate and right-wing crime. However, we hypothesized that they should be in the same direction, that is, unemployment rate may be positively linked with right-wing crime and proportion of foreigners negatively with AfD electoral success (Hypothesis 1c).

In a second set of hypotheses, we predicted specific links of socio-structural factors with survey data. As such “cross-level” links are not commonly theorized or researched, we based our hypotheses on a general reading of the literature on intergroup contact (Allport, 1954; Pettigrew, 1998; Pettigrew and Tropp, 2006) and deprivation theory (Runciman, 1966; Major, 1994; Pettigrew and Meertens, 1995) and predicted psychological perceptions to be linked with the corresponding socio-structural parameters in the suitable fashion. More specifically, we expected proportion of foreigners to be linked with intergroup contact (Hypothesis 2a) and unemployment rate with perceptions of fraternal deprivation (Hypothesis 2b). Both should in turn be correlated with extreme right-wing attitudes (Hypothesis 2c) that should be predictive of both outcome variables (Hypothesis 2d).

We made no predictions regarding intercorrelations of socio-structural predictors or outcomes. However, regarding far right electoral support and right-wing crime, we hypothesized that they might be correlated because they are facilitated by similar socio-structural as well as psychological factors.

## Materials and Methods

In a first step, we combined data from three independent and official sources: socio-structural data for the year 2016 provided by the German office for statistics, 2017 election results available through the Federal Election Commissioner, and 6,354 reports of crimes targeting refugees and their homes filed as “politically motivated crime, right-wing” by the police between 2015 and 2017 that were collected in an overview (Federal Government, 2016, 2017, 2018). In a second step, we added survey data from a representative sample drawn in 2016 into the data set.^3^ We shall now briefly describe each of our data sources in turn and how they were combined before analyzing their interrelations more systematically.

### Socio-Structural Data

Official numbers of residents per municipality were available through the German office for statistics along with other information on absolute numbers regarding legal status (unemployed persons and foreigners). We used these numbers to generate unemployment rates for June 2016 (ranging from 1.2 – 14.7%) and foreigners per municipality for 2016 (ranging from 1.96 – 33.91%).

### Election Results

Results for the 2017 national elections are available from the Federal Election Commissioner along with total valid votes per municipality. We computed electoral success for the AfD as one dependent variable by dividing valid AfD votes by total valid votes per municipality (ranging from 4.94 – 35.46%).

### Right-Wing Hate Crimes

Our second dependent variable was the number of right-wing attacks and crime targeting refugees and their homes reported to police within municipalities in 2017. We compiled an overview of 2.211 such incidents based on police statistics. The numbers of attacks targeting refugees and their homes are published by the federal Government in so called *Antworten der Bundesregierung* (official replies by the Federal Government to requests by parliamentarians and parties). These are special reports filed by the Government answering inquiries officially requested by parliamentary parties or MPs covering various political issues. The statistics on hate crimes targeting refugees and their homes were published quarterly and in a final version by the government in response to members of parliamentary party *Die Linke* (The Left). Crimes reported ranged in severity from right-wing graffiti and dissemination of propaganda to defamation and harassment all the way to assault, bomb attacks, and homicide. The crimes had been categorized by the Government as “politically motivated crime, right-wing” linking them directly to the “refugee subject matter” (Federal Government, 2018). Records included running number, date, location, federal state as well as the most severe reported offense. One sample line reads “268, 19.10.2017, Erftstadt, NW (Federal state of North Rhine-Westphalia), Schwere Brandstiftung §306a StGB (severe case of arson)” (Federal Government, 2018, p. 18).

Two independent coders placed each reported crime within the respective municipality based on where it had been recorded. The index of right-wing crimes reported in 2017 ranged from 0 (e.g., in Bottrop) up to 58 in Chemnitz. As municipalities vary considerably in their numbers of residents and in order to yield similar ranges for this index as for the other indices, we used number of right-wing crimes targeting refugees and their homes reported per 10,000 inhabitants for the analyses to be reported below.

### Survey Data

A representative sample of 2,008 German participants was surveyed in standardized telephone interviews that were conducted by a professional survey institute between June and August 2016. The survey covered measures for cross-group friendships, perceived economic threat, and attitudes toward various political issues including extreme right-wing attitudes that we will focus on in this paper and describe below in more detail (see Zick et al., 2016). To ascertain representativeness of the sample, telephone numbers were randomly generated, and the last-birthday method was used to randomly select participants within households. As 25% of the participants were contacted via mobile phone numbers and we needed to assign data to municipality- (Kreis-) level by city-prefix, we only used data from those *N* = 1,506 participants that were contacted by landline.

Both sub-samples differed somewhat in terms of age and gender, with more younger (*M* = 46.10, *SD* = 16.93), *t*(1896) = 8.75, *p* < 0.001, and more male participants (55%), χ^2^ (*N* = 1917, *df* = 1) = 18.68, *p* < 0.001, in the mobile-only sub-sample than in the landline-only sub-sample. Crucially, however, both samples did not differ in terms of level of education and those measures that were of interest for our analyses.

The resulting landline-only sample was *M* = 53.98 years old (*SD* = 16.93), with slightly more female than male participants (54.4%). Level of education was slightly skewed with 53.6% of the sample holding a university or technical degree, 28% reporting some secondary school-leaving certificate, and 12.8% reporting no degree at all. Monthly household net income was 20.2% “less than 2,000 EUR”, 19.9% “more than 2,000 but less than 3,000 EUR”, 13.6% “more than 3,000 but less than 4,000 EUR”, and 19.8% “4,000 EUR and more”.

#### Perceived Collective Deprivation

*Perceived collective deprivation* was measured with the item “How would you judge the economic situation of Germans compared with foreigners living here?” and answers ranging from 1 “very good” to 5 “very bad”.

#### Intergroup Contact

*Intergroup contact* was measured with the item “How many of your friends or close acquaintances have a migration background?” with answers ranging from 1 “none” to 4 “very many”.

#### Extreme Right-Wing Attitudes

*Extreme right-wing attitudes* were measured using seven items on a scale ranging from 1 “completely false” to 5 “completely true”. Items included statements such as “I can understand that some citizens resist forcefully against homes for asylum seekers” and “No one can expect for me to live next to a home for asylum seekers” (Cronbach’s alpha = 0.83; see section “Supplementary Material” for the full scale).

As the analyses reported in the current study were based on secondary analyses of official sources and on data previously collected in a survey, ethics approval was not required as per applicable institutional and national guidelines and regulations. For the survey data used in our analyses, informed consent of the participants was implied through survey completion. Participation in the survey was completely voluntary and anonymous and participants were free to withdraw from the survey at any time without incurring any penalties.

## Results

### Preliminary Analyses

Means, standard deviations, and zero-order correlations are shown in Table 1 for all socio-structural variables on municipality level and in Table 2 for all psychological variables on both individual (lower triangle) and municipality level (upper triangle).^4^ As can be seen, for the latter, means, standard deviations, and correlational patterns did not differ significantly between individual and municipality level. We used psychological measures aggregated on municipality level for the current analyses to link them with socio-structural variables. On the one hand, it should be kept in mind that measures varied in range due to their different sources. On the other hand, it seems noteworthy that most of them were systematically linked *despite* their different sources even after correcting for skew or using non-parametric test procedures (see Footnote 4). Extreme right-wing attitudes, for example, were significantly and negatively correlated with proportion of foreigners, *r*(346) = −0.13, *p* = 0.02, but positively with far right electoral support, *r*(345) = 0.19, *p* < 0.001, and with right-wing crimes reported in the respective municipalities in the subsequent year, *r*(346) = 0.12, *p* = 0.03. While these correlations are small in magnitude, they were all significant and in the expected direction. Recall that attitudes were measured in an independent telephone survey. These were also correlated with our dependent variables as can be seen from Table 1.

**TABLE 1:** Means, standard deviations, and zero-order correlations for socio-structural data on municipality level.

	***M* (*SD*)**	**(2)**	**(3)**	**(4)**
(1) Unemployment rate	0.06 (0.03)	0.03	0.25^∗∗∗^	0.33^∗∗∗^
(2) Proportion of foreigners	0.10 (0.05)		−0.41^∗∗∗^	−0.29^∗∗∗^
(3) AfD electoral support	0.13 (0.05)			0.50^∗∗∗^
(4) Right-wing crimes	0.29 (0.37)			

*(1) through (3) are per 100, and (4) per 10,000 inhabitants. ^∗∗∗^*p* < 0.001.*

**TABLE 2:** Means, standard deviations, and zero-order correlations for data on individual and municipality level.

	***M* (*SD*)**				
	**Individual**	**Municipality**	**(1)**	**(2)**	**(3)**
(1) Collective Deprivation	2.35 (0.90)	2.35 (0.66)		−0.12^∗^	0.42^∗∗^
(2) Contact	2.17 (0.84)	2.07 (0.59)	−0.06^∗∗^		−0.25^∗∗^
(3) Extreme Right-wing Attitudes	2.34 (0.95)	2.39 (0.66)	0.30^∗∗^	−0.23^∗∗^	

*Individual level correlations in lower and municipality level correlations in upper triangle. ^∗^*p* < 0.05, ^∗∗^*p* < 0.01.*

**FIGURE 1:**
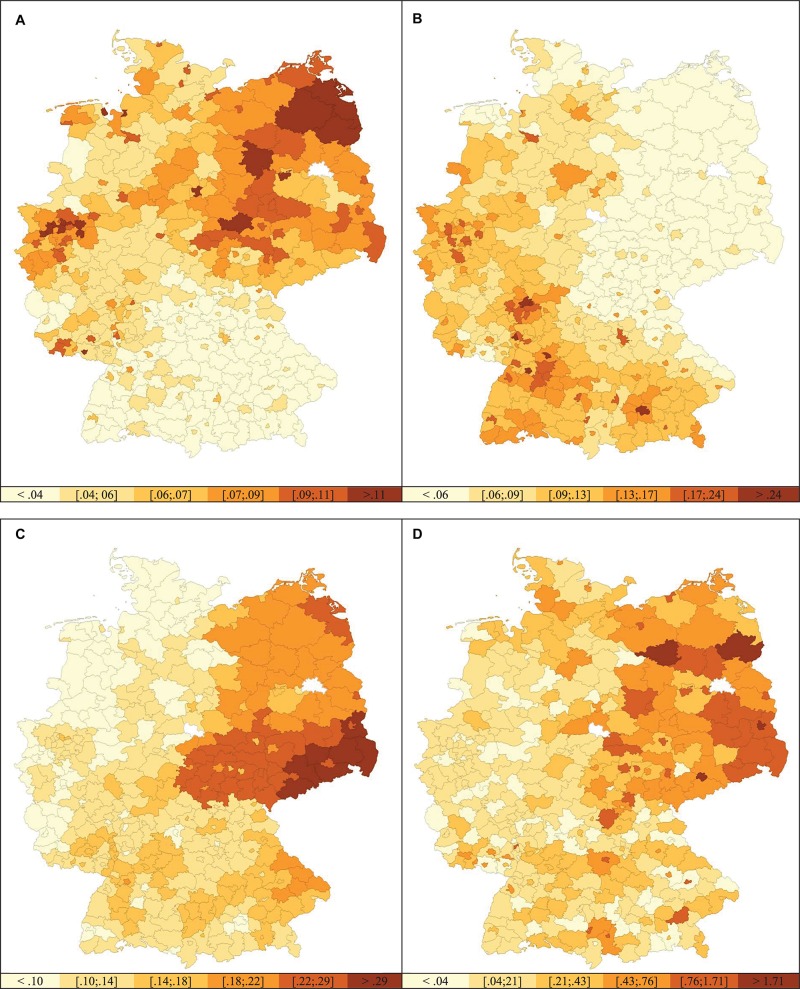
Natural breaks maps of Germany illustrating spatial distributions of socio-structural and outcome variables: **(A)** unemployment rate, **(B)** proportion of foreigners, **(C)** far right electoral support, **(D)** and right-wing crime. Berlin was excluded from the analyses.

Local unemployment rates in June 2016, for example, while unrelated to local proportions of foreigners in the same year, were correlated with both local far right electoral support, *r*(397) = 0.25, *p* < 0.001, and right-wing crimes reported in the respective municipalities 1 year later, *r*(398) = 0.40, *p* < 0.001. As one final correlational result, both dependent variables, far right electoral support and right-wing crimes reported were linked substantially and positively, *r*(399) = 0.50, *p* < 0.001. Spatial distributions of socio-structural and outcome variables are illustrated in Figure 1.

Figure 1 not only illustrates socio-structural variations across municipalities – unemployment rates tend to be highest in the East and the Ruhr area and lowest in the South of Germany, proportions of foreigners per municipality fall within the lowest category in almost all Eastern German municipalities. There is also a striking East-West difference regarding both outcome variables: far right electoral support was highest in the Eastern and Southern municipalities and the proportion of right-wing crime seems to be higher in these areas, too. These differences also show empirically with significantly higher unemployment rates (*M*_*East*_ = 0.08, *SD* = 0.02 vs. *M*_*West*_ = 0.05, *SD* = 0.02), lower proportions of foreigners (*M*_*East*_ = 0.04, *SD* = 0.02 vs. *M*_*West*_ = 0.11, *SD* = 0.05), higher far right electoral support (*M*_*East*_ = 0.22, *SD* = 0.05 vs. *M*_*West*_ = 0.11, *SD* = 0.03), and more right-wing crime reported per municipality on average (*M*_*East*_ = 0.75, *SD* = 0.55 vs. *M*_*West*_ = 0.18, *SD* = 0.20), *t*(399) = 9.26, *t*(322) = 20.56, *t*(87) = 18.94, and *t*(79) = 8.80, respectively, all *p*s < 0.001.^5^ As East-West differences regarding attitudes and behavior toward minority groups in Germany have been demonstrated before (Wagner et al., 2003; Benček and Strasheim, 2016; cf. Czymara and Schmidt-Catran, 2016), and may distort the results of the following analyses, we decided to account for East-West differences where appropriate and feasible.

### Testing the Proposed Model

The correlational analyses reported above lend some initial support to our proposed model. However, they leave open the issue of shared variance in predicting the outcome variables as well as the question of how much predictive value – if any at all – is added above and beyond harder socio-structural variables through softer variables such as extreme right-wing attitudes measured in a telephone survey. In a first set of analyses, we consequently addressed the issue of shared variance. Crucially, in assuming random error, conventional statistical models do not account for shared variance due to geographical proximity or spatial auto-correlation. We addressed this issue in a second set of analyses using GWR analyses. In a third and final set of analyses, we accounted for and explored the differences between East and West Germany reported above.

#### Controlling for Potential Overlap on Predictor and Criterion Sides

**FIGURE 2:**
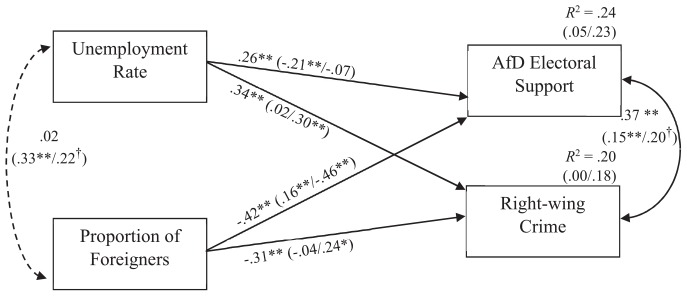
Path model of socio-structural correlates of far right electoral support and political hate crimes in Germany. Standardized path coefficients for overall analyses; West and East Germany separately in brackets (West/East); error terms are not displayed for the sake of clarity. ^∗∗^*p* < 0.01, ^∗^*p* < 0.05, ^†^*p* < 0.10.

First, we performed path analyses to account for potential overlap both on predictor and criterion sides and to test how much predictive value our psychological survey measures would add. Path analyses were performed using AMOS 24.0 and maximum likelihood estimation. The model was composed in such a way that the socio-structural variables, unemployment rate and proportion of foreigners correlated with far right electoral support and right-wing crime (Figure 2). All four paths remained significant and almost unchanged when compared to the zero-order correlations: Proportion of foreigners per municipality on the one hand were negatively linked with both right-wing crimes reported, β = −0.31, and even more strongly with far right electoral support 1 year later, β = −0.42. Unemployment rate on the other hand was linked with both AfD electoral success, β = 0.26, and even more strongly with right-wing crimes reported, β = 0.34 1 year later (all *p*s < 0.001). Taken together, both socio-structural predictors explained 20% and 24% of variance in right-wing crimes reported and AfD electoral support, respectively. To correct for skew in the data, we also performed bootstrapping analyses using 5000 bootstrap resamples and bias-corrected 95% confidence intervals (CIs). As none of the resulting CIs included zero, these analyses further supported the model.

**FIGURE 3:**
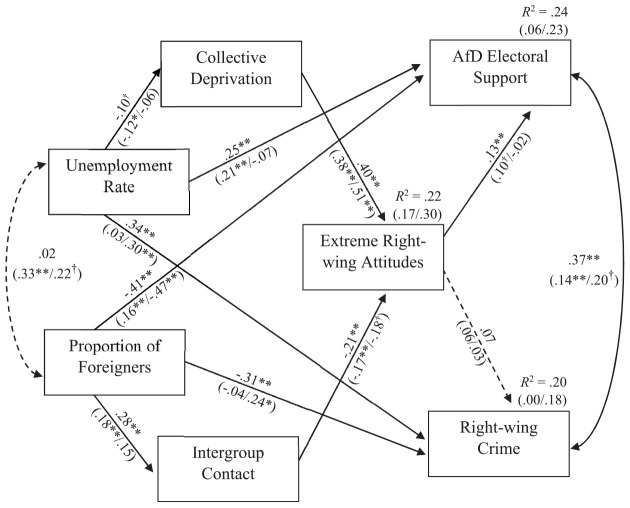
Path model of socio-structural and psychological correlates of right-wing electoral support and political hate crimes in Germany. Standardized path coefficients for overall analyses; West and East Germany separately in brackets (West/East); error terms are not displayed for the sake of clarity. ^∗∗^*p* < 0.01, ^∗^*p* < 0.05, ^†^*p* < 0.10.

We then introduced the three psychological variables, collective deprivation, contact, and extreme right-wing attitudes, into the model (Figure 3). A first saturated model included paths between all constructs and, expectedly, fitted the data perfectly. We then computed a second model where a total of eight paths that we had not predicted were set to zero. Specifically, these were paths from socio-structural variables to psychologically incongruent constructs (i.e., paths from proportion of foreigners to collective deprivation and from unemployment rate to contact) and paths that we predicted to be zero because we assumed the psychological contribution to be mediated through extreme right-wing attitudes (i.e., paths from socio-structural variables to extreme right-wing attitudes and paths from contact and collective deprivation to both outcome variables, respectively). As this second model was nested within the previous one, we compared both and concluded that the second explained the data equally well, Δχ^2^ (*df* = 8) = 12.21, *p* = 0.14. It seems noteworthy that, as predicted, the deleted paths were generally statistically non-significant (*p*s > 0.10) except for the path from unemployment rate to contact (β = 0.12, *p* = 0.02). The resulting model is displayed in Figure 2. It had a good fit with the data, χ^2^ (*N* = 400, *df* = 8) = 15.57, *p* = 0.05, CFI = 0.98, RMSEA = 0.05, PCLOSE = 0.47 (MacCallum et al., 1996; Hu and Bentler, 1999).

The original pattern remained almost unchanged when psychological variables were included into the full model. Extreme right-wing attitudes which had been significantly correlated with both outcome variables only retained a weak but reliable link with far right electoral support, β = 0.13, *p* = 0.01, but were not significantly linked with right-wing crime reported, β = 0.07, *p* = 0.16. Explained variance in both dependent variables remained unchanged.^6^

#### Accounting for Shared Variance Due to Geographical Proximity

As briefly mentioned above, the analyses reported thus far neglect spatial auto-correlation or shared variance due to mere geographical proximity. Such shared variance may be due to common exposure of the observed variables to unobserved confounders and can create problems for conventional statistical models that assume random error (see Fotheringham et al., 2002; Teney, 2012). We performed a series of GWR analyses using spatial error models based on maximum likelihood estimation and queen contiguity weights in GeoDA (Version 1.12; Anselin et al., 2006; GeoDa, 2019) to address the issue of spatial auto-correlation. As can be seen from Table 3, results from these analyses replicated the results reported above in showing that unemployment was linked positively with both far right electoral support and right-wing crime while proportion of foreigners was linked negatively with both outcomes. Regarding the survey measures, the GWR analyses confirmed that they had weak but significant links with far right electoral success above and beyond socio-structural variables – the respective model was superior to that without survey measures according to the Akaike Information Criterion (AIC).^7^ Survey measures did not contribute significantly to the explained variance in right-wing crime as the outcome variable, however. In fact, that model became significantly worse according to the associated AIC. It seems noteworthy that the Lambda-coefficients were significant across all analyses: there was strong support for spatially correlated errors. Accordingly, the GWR models explained substantially more variance in both far right electoral support (*R*^2^ = 0.83) and right-wing crime (*R*^2^ = 0.38) than conventional regression models.

**TABLE 3:** Geographically weighted regression (GWR) results for socio-structural correlates of far right electoral support (top panel) and political hate crimes in Germany (bottom panel).

	**Coeff.**	**S.E.**	***z***	**Coeff.**	**S.E.**	***z***
**AfD electoral support**						
Constant	0.12	0.01	12.50^∗∗^	0.14	0.01	12.60^∗∗^
Lambda	0.86	0.03	33.05^∗∗^	0.85	0.03	29.88^∗∗^
Unemployment rate	0.47	0.08	5.76^∗∗^	0.40	0.09	4.56^∗∗^
Proportion of foreigners	–0.23	0.04	5.50^∗∗^	–0.22	0.04	5.02^∗∗^
Collective deprivation				–0.01	0.001	3.31^∗∗^
Contact				< 0.001	0.002	<1
Extreme right-wing attitudes				0.003	0.002	2.04^∗^
*R*^2^	0.83			0.83		
AIC	−1818.54			−1559.36		
**Right-wing Crime**						
Constant	0.11	0.06	1.63	0.05	0.12	<1
Lambda	0.59	0.05	11.68^∗∗^	0.58	0.05	10.96^∗∗^
Unemployment rate	5.72	0.86	6.62^∗∗^	5.90	0.94	6.28^∗∗^
Proportion of foreigners	–1.11	0.44	2.53^∗^	–1.39	0.48	2.93^∗∗^
Collective deprivation				0.02	0.03	<1
Contact				0.03	0.03	1.01
Extreme right-wing attitudes				–0.01	0.03	<1
*R*^2^	0.38			0.39		
AIC	189.09			172.94		

*^∗^*p* < 0.05, ^∗∗^*p* < 0.01.*

#### Exploring East-West Differences

As we had observed considerable differences for all variables of interest, we controlled for and explored the differences between East and West Germany in a third and final set of analyses. While accounting for spatial auto-correlation in GWR analyses and East-West differences through dummy-coding, we also included interaction terms with both socio-structural variables. When predicting far right electoral support, we found a significant interaction of proportion of foreigners and the East-West dummy variable, *z* = 7.56, *p* < 0.001. Similarly, when predicting right wing-crime, we found interactions with the East-West dummy variable for both unemployment rate, *z* = 4.37, *p* < 0.001, and proportion of foreigners, *z* = 4.20, *p* < 0.001.

Following up on these results, we analyzed the data for municipalities in East and West Germany separately. For far right electoral support, both socio-structural factors retained significant links with the outcome: unemployment rate retained links of similar magnitude in the West, *b* = 0.36, *z* = 5.01, compared with the East, *b* = 0.44, *z* = 2.65, *p*s < 0.01. Proportion of foreigners, however, was linked much more strongly with the outcome in the East, *b* = −1.19, *z* = 7.92, *p* < 0.001 than in the West, *b* = −0.10, *z* = 2.76, *p* = 0.01.

For right-wing crime, when analyzing East and West German municipalities separately, proportion of foreigners was no longer significantly linked with the outcome in West Germany, *z* < 1, and unemployment rate still retained a positive but much weaker link, *b* = 1.02, *z* = 1.70, *p* = 0.09. In East Germany, however, both socio-structural factors were still linked with the outcome: unemployment rate, *b* = 8.20, *z* = 2.64, as well as proportion of foreigners, *b* = 8.48, *z* = 2.77, *p*s < 0.01, were correlated with right-wing crime significantly and *positively*.

Bootstrap analyses with 5000 resamples and bias-corrected 95% CIs generally replicated these results with some exceptions: CIs for the paths from socio-structural factors to far right electoral support did not include zero with the exception of the path from unemployment rate to far right electoral support. For right-wing crime, only those paths in East Germany were reliable according to these analyses and did not include zero while both paths in West Germany did. In a sense then this pattern of results was similar but more pronounced than that of the GWR analyses.

## Discussion

The present study systematically combined data on socio-structural variables (unemployment rates and proportion of foreigners by municipality) with self-reported attitudes and official data on actual behavior (police records on right-wing hate crime and far right electoral support in the German federal elections). In doing so, we tried to approximate what could be referred to as a “climate of hate” – a bundle of objective as well as more subjective or psychological variables all of which contribute to a social norm or a perception of such a norm that facilitates hostile behaviors toward outgroups. One central notion of the current work is, consequently, that more conventional but potentially exclusionary behaviors such as far right voting on the one hand and more extreme behaviors such as right-wing hate crimes targeting refugees on the other hand should co-occur because they are facilitated by similar factors.

### Correlates of Behavioral Outcomes and Local Variation

While unrelated with each other, both socio-structural factors were linked with both outcome variables in a systematic fashion: First, overall, the local proportion of foreigners was negatively correlated with relative number of hate crimes, and more strongly so with far right electoral support in municipalities. Second, unemployment rate was positively linked with far right electoral support, and more strongly so with relative number of hate crimes reported. When examining these links for East and West German municipalities separately, the pattern remained similar for correlates of far right electoral support. However, the pattern changed drastically for right-wing crime: While unemployment rate was still positively linked with right-wing crime across Germany (but substantially weaker so in Western municipalities), proportion of foreigners was no longer linked with the same outcome in West German municipalities. Contrary to what we had expected, in East German municipalities, this relation even reversed and proportion of foreigners was significantly and positively linked with right-wing crime reported. This pattern of results can be interpreted as an artificial overall negative correlation between proportion of foreigners and reported right-wing crime that can be attributed to the mean differences in measures between East and West Germany. With regard to the two behavioral outcomes, this local variation in their correlates may provide preliminary evidence for related but contrary underlying motives: While one seems to be driven by contact-logic (more contact opportunities, less far right electoral support; e.g., Allport, 1954), the other seems to be more in line with group threat-logic (more foreigners, more right-wing crime; e.g., Teney, 2012). Methodologically, however, this result emphasizes the importance of considering local variation of the phenomena that are being investigated.

Attitudinal variables measured in a representative survey were also linked with socio-structural variables in the predicted direction, that is, intergroup contact correlated positively with local proportion of foreigners, thus replicating a host of previous research (Wagner et al., 2003, 2008; Semyonov et al., 2004). Furthermore, collective deprivation correlated positively with unemployment rates, and both, intergroup contact and collective deprivation predicted extreme right-wing attitudes. However, these predictors’ contribution to the explained variance in outcome variables above and beyond socio-structural variables was neglectable. We will address the limitations of the present research in more detail further below after discussing potential implications that can be drawn.

### Identifying Areas at Risk for Right-Wing Extremism Through Contextual Indicators

In order to reduce or prevent political extremism, a first step can be to identify and monitor areas that are at particularly high risk of violent hate crimes. So far, monitoring instruments for such hate crimes have been scarce or non-existent (Benček and Strasheim, 2016; but see ProAsyl, 2017). We argue that high risk areas can still be identified based on contextual factors that are reliable correlates of actual violent extremism and that such correlates can be found in the neighboring research field on far right electoral support. For example, some previous research has predicted incidents of violent right-wing attacks from analyses of public discourse (Koopmans and Olzak, 2004) or from social media data (Müller and Schwarz, 2018). Scholars in the social sciences also seem to agree that there is merit in collecting data on political attitudes including attitudes toward outgroups in order to identify risks or at least shed light on psychological processes that may turn prejudice into violence (e.g., Wagner et al., 2003). All in all, the notion that contextual factors can be useful in identifying high risk areas is not new and two factors – unemployment rate and proportion of foreigners in a given local context – have been particularly well studied (Semyonov et al., 2004; Wagner et al., 2008). In his review of research on far right electoral success, Golder (2016) has systematized these contextual factors into “economic grievances” and “cultural grievances”. In discussing the global rise of the far right, some have argued that it is more about culture than economics (e.g., Norris and Inglehart, 2019), others that it is more about economics than culture (Judis, 2018). And even others have argued that such a binary distinction between economic and cultural grievances is “far too simplistic and glosses over the way in which concerns about culture and economics can, and often do, interact” (Eatwell and Goodwin, 2018, p. xxiv). We argue that much can be learned from investigating far right electoral support when studying right-wing hate crime and that both fields can benefit from each other. Based on the current study, we conclude that violent right-wing hate crime is particularly likely in areas with high unemployment rates (as is far right electoral support) and a high proportion of foreigners (contrary to far right electoral support) but that this latter correlate may vary locally.

This finding is somewhat contradictory to intergroup contact theory (Pettigrew, 1998; Wagner et al., 2006) but well in line with the group threat hypothesis and the intuition that, in order for hate crimes to occur, the target outgroup needs to be present. Diversity, while increasing community resilience against far right agitation through contact opportunities (Allport, 1954; Pettigrew and Tropp, 2006) may ironically increase the risk of right-wing crime in the same area.

Finally, far right electoral support was so strongly correlated with relative number of right-wing hate crimes that it might be considered an additional indicator for areas that are at high risk for right-wing extremism. In other words, our results seem to be supportive of the notion that far right electoral support is not only an indicator, but actually part of the social climate of hate that facilitates right-wing violence.

### Limitations and Future Research

There are limitations of the current study, some of them due to its overall cross-sectional design or the nature of the data we use. First, we report correlational data that do not allow for causal inferences. While the basic premise of the current work does not necessarily hinge on causal relationships between the constructs but is merely to show that two behavioral outcomes are linked with the same socio-structural correlates and co-occur systematically in certain areas, the variables we used as predictors were all measured 1 year prior to the variables we used as criteria. We would therefore argue that the analyses reported are at least to some extent suggestive of the predictive value of socio-structural and survey data for future outcomes. The distinction between causality and correlation, however, is crucial especially for policy makers and practitioners and future research using longitudinal designs might tackle the issue of causality more convincingly.

Second, from a methodological point of view, the compatibility of our measures especially at the interface of socio-structural and survey data may be open to criticism. More specifically, one could argue that perceived competition on the labor market would be more compatible with local unemployment than perceived collective deprivation. Also, the use of single item-measures is problematic. The weak explanatory performance of attitudinal variables, in other words, may then be due to issues of validity and reliability. However, empirically, we think the measures we used tap into the respective constructs – they do in fact correlate with socio-structural variables in the expected fashion (i.e., proportion of foreigners correlates with contact and unemployment correlates with perceptions of deprivation). While more elaborate data on the psychological level including longer scales would certainly be desirable, such data were not available for the analyses presented in this contribution. Our analysis may thus serve as a proof of principle and hopefully inspire future research to link socio-structural data with survey data and attitudinal as well as actual behavioral outcomes. Such research could also take context into account more systematically by studying specific other European countries experiencing an increase in far right electoral support, such as, Hungary (Palonen, 2009), Italy (Verbeek and Zaslove, 2016), or The Netherlands (Otjes and Louwerse, 2013) or by comparing far right electoral support and the prevalence of hate-crime in countries across Europe (e.g., Lubbers et al., 2002).

Furthermore, future research might benefit from qualitative or mixed-methods approaches, ideally in a longitudinal design to examine regional specifics and developments in those municipalities and areas most affected by a climate of hate. It seems fruitful to investigate those contextual factors qualitatively, social constellations, and regional specifics that facilitate a climate of hate in identified risk areas to draw conclusions on how to strengthen community resilience against extreme right-wing behavior. Methodically, some studies already follow this approach, taking closer looks at intergroup relations on a small-scale level (Bynner, 2017) or at the “normalization” of anti-refugee sentiments in everyday life of a medium sized town (Kurtenbach, 2018).

### Conclusion

The results of the current study can be placed within the wider research fields on far right electoral support (e.g., Golder, 2016; Eatwell and Goodwin, 2018) and the prevalence of hate-crime in countries across the world. Understanding both phenomena as partly connected may have important implications for future research, both basic and applied, as well as for politics and practice. Practitioners and policy-makers may find them useful in developing effective strategies to prevent or at least reduce right-wing extremism by identifying high risk areas. Diverse communities should be more resilient against far right agitation whereas areas with little heterogeneity and high unemployment rates are susceptible for a general climate of hate. A decentralized housing policy for newcomers like refugees may thus decrease far right support but also increase the risk for right-wing crime.

Our final conclusion relates to the added value of survey data in identifying high risk areas. We believe that attitudinal data and surveys will continue to contribute invaluable insights into the processes of prejudice, discrimination, and radicalization. However, our analysis and its results might also serve as a cautionary note: Measures collected in a representative survey were generally linked with socio-structural indicators in the predicted pattern. Self-reported extreme right-wing attitudes were even correlated with actual voting behavior in municipalities 1 year later. While this is good news for attitude research in general and social scientists in particular, the bad news is that the incremental predictive value of these survey data above and beyond socio-structural indicators was neglectable.

## Data Availability Statement

The datasets generated for this study are available on request to the corresponding author.

## Ethics Statement

Ethical review and approval was not required for the study on human participants in accordance with the local legislation and institutional requirements. Written informed consent for participation was not required for this study in accordance with the national legislation and the institutional requirements.

## Author Contributions

JR and YR planned the study, collected, compiled the data set, and drafted the manuscript together with JH. JR analyzed the data. AZ provided crucial comments and changes to all parts of the manuscript. All authors approved the submission of the final version of the manuscript.

## Conflict of Interest

The authors declare that the research was conducted in the absence of any commercial or financial relationships that could be construed as a potential conflict of interest.

## Footnotes

1 We refer to the far right party family following Golder (2016) where a more elaborate discussion and distinction between radicalism, populism, extremism, and nationalism within the far right can be found.

2 Officially, there is no hate crime legislation in the German Criminal Code. However, the legal equivalent is called, politisch motivierte Kriminalität rechts “(PMK-r), which is officially defined as crime “against a person based on political attitude, nationality, ethnicity, race, skin color, religion, world view, origin, or physical appearance, disability, sexual orientation or societal status (so called “hate crimes”)” (German Ministry of the Interior Building and Community, 2019).

3 Some data sources differentiated between East or West Berlin while others did not. We therefore decided, following previous research (e.g., Wagner et al., 2003) not to include Berlin in our analyses. We note, however, that the pattern of results did not change substantially when analyses were re-run including Berlin as a whole.

4 To correct for positive skew in the data, analyses were re-run using non-parametric tests or bootstrapping analyses where appropriate. As they yielded the same pattern of results that is described here and in the following sections, we report results based on the original data and parametric tests.

5 The pattern of results remained the same when applying non-parametric Mann–Whitney tests (see section “Supplementary Material” for a summary of these results).

6 An argument could be made that collective deprivation is not causally prior to extreme right-wing attitudes but that it is in fact part of or even an outcome of extreme right-wing attitudes. We therefore tested alternative models including (a) a reverse path from extreme right-wing attitudes to collective deprivation and (b) a bi-directional path between both constructs. Both models fitted the data descriptively better than the original model that we had hypothesized. It therefore seems worthwhile to note again that we are not testing any causal mechanisms based on the correlational data reported.

7 As a rule of thumb, an AIC difference of <2 indicates no meaningful discrepancy between models; a difference between 4 and 7 indicates considerable evidence that the model with the lower AIC is better, and a difference of >10 indicates substantial support for the model with the lower AIC (Burnham and Anderson, 2002).
