# Supplementary material for: Climate of Hate: Similar Correlates of Far Right Electoral Support and Right-Wing Hate Crimes in Germany
Source: Front Psychol. 2019 Oct 18;10:2328. doi: 10.3389/fpsyg.2019.02328 (PMC6813724; doi:10.3389/fpsyg.2019.02328)
Supplement: Supplementary file 1 [file Data_Sheet_1.docx]

Extreme right-wing attitudes scale

| Item | *M* (*SD*) | *r*_IT_ |
| --- | --- | --- |
| I can understand that some citizens resist forcefully against homes for asylum seekers | 1.33 (0.85) | .39 |
| We should not integrate foreigners here but bring them back to their home countries and support them there. | 2.51 (1.41) | .57 |
| The citizens’ anger at immigration is absolutely understandable. | 2.33 (1.36) | .68 |
| No one can expect for me to live next to a home for asylum seekers | 1.80 (1.21) | .54 |
| Foreign influences on our culture have to be restricted to a reasonable degree. | 2.93 (1.56) | .61 |
| You cannot say anything bad about foreigners in Germany without being insulted as a racist. | 3.25 (1.51) | .59 |
| In Germany, you can no longer express your opinion without getting into trouble. | 2.21 (1.43) | .61 |

Means, standard deviations, and zero-order correlations for socio-structural data on municipality level (square-root transformed data).

|  | *M* (*SD*) | (2) | (3) | (4) |
| --- | --- | --- | --- | --- |
| (1) Unemployment rate | .23 (0.05) | -.04 | .22*** | .30*** |
| (2) Proportion of foreigners | .30 (0.08) |  | -.47*** | -.34*** |
| (3) AfD electoral support | .36 (0.07) |  |  | .47*** |
| (4) Right-wing crimes | .45 (0.30) |  |  |  |

*** *p* < .001

Spearman correlations for socio-structural data on municipality level.

|  | (2) | (3) | (4) |
| --- | --- | --- | --- |
| (1) Unemployment rate | -.08 | .12* | .26*** |
| (2) Proportion of foreigners |  | -.35*** | -.38*** |
| (3) AfD electoral support |  |  | .40*** |
| (4) Right-wing crimes |  |  |  |

* *p* < .05

*** *p* < .001

Spearman correlations for data on individual and municipality level.

|  |  |  |  |
| --- | --- | --- | --- |
|  | (1) | (2) | (3) |
| (1) Collective Deprivation |  | -.10^†^ | .34** |
| (2) Contact | -.06** |  | -.23** |
| (3) Extreme Right-wing Attitudes | .28* | -.24** |  |

*Note*. Individual level correlations in lower and municipality level correlations in upper triangle.

^†^ *p* = .06, * *p* < .05, ** *p* < .01

Mann-Whitney test results for East-West differences.

|  | *Mdn*_West_ | *Mdn*_East_ | *U* | *z* |
| --- | --- | --- | --- | --- |
| Unemployment rate | .045 | .074 | 4195 | 8.97*** |
| Proportion of foreigners | .097 | .035 | 1246 | 12.20*** |
| AfD electoral support | .111 | .218 | 402 | 13.13*** |
| Right-wing crimes | .138 | .632 | 2275 | 11.08*** |

*** *p* < .01

Quantile maps of Germany illustrating spatial distributions of socio-structural and outcome variables: unemployment rate (a), proportion of foreigners (b), far right electoral support (c), and right-wing crime (d).

| a 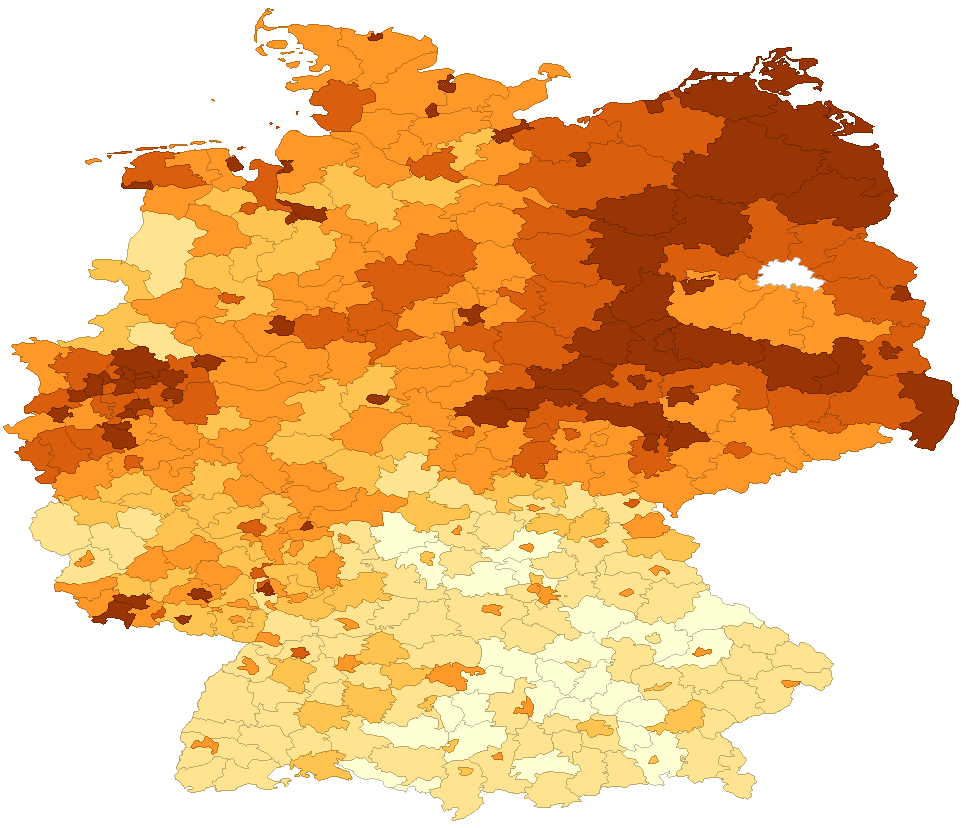 | | | | | | b 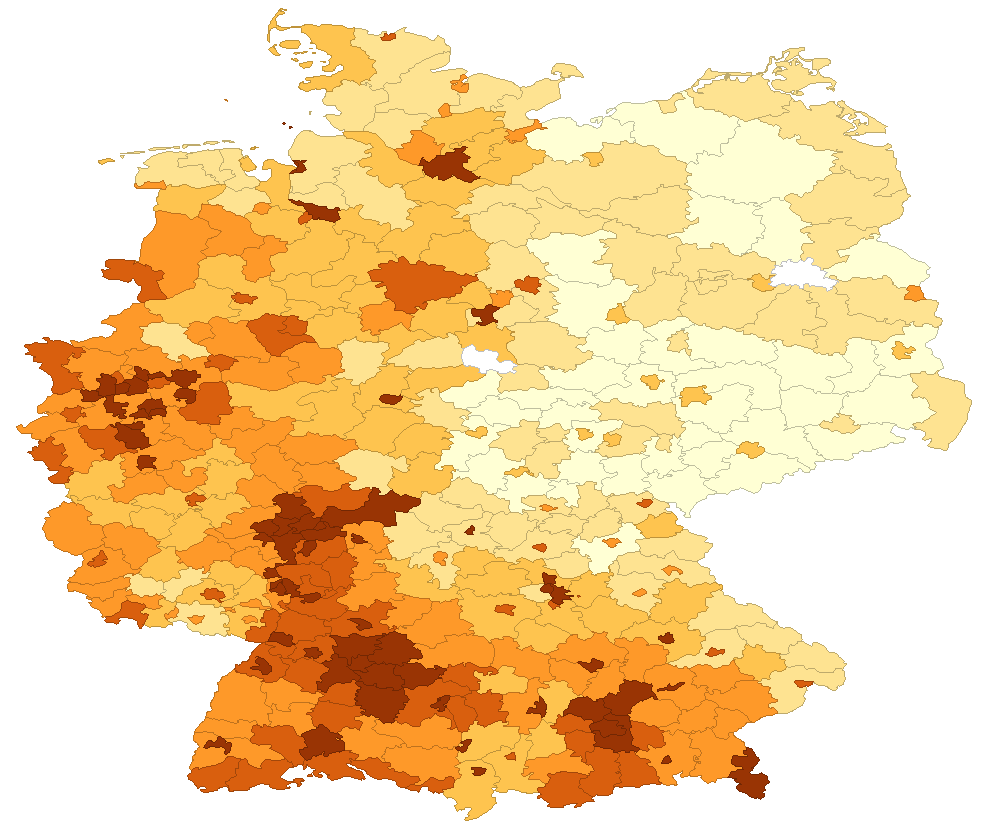 | | | | | |
| --- | --- | --- | --- | --- | --- | --- | --- | --- | --- | --- | --- |
| < .02 | 03 | .04 | [.05;.06] | [.07;.08] | >.09 | < .03 | [.04;.06] | [.07;.08] | [.09;.11] | [.12;.14] | > .15 |
|  | | | | | | | | | | | |
| c 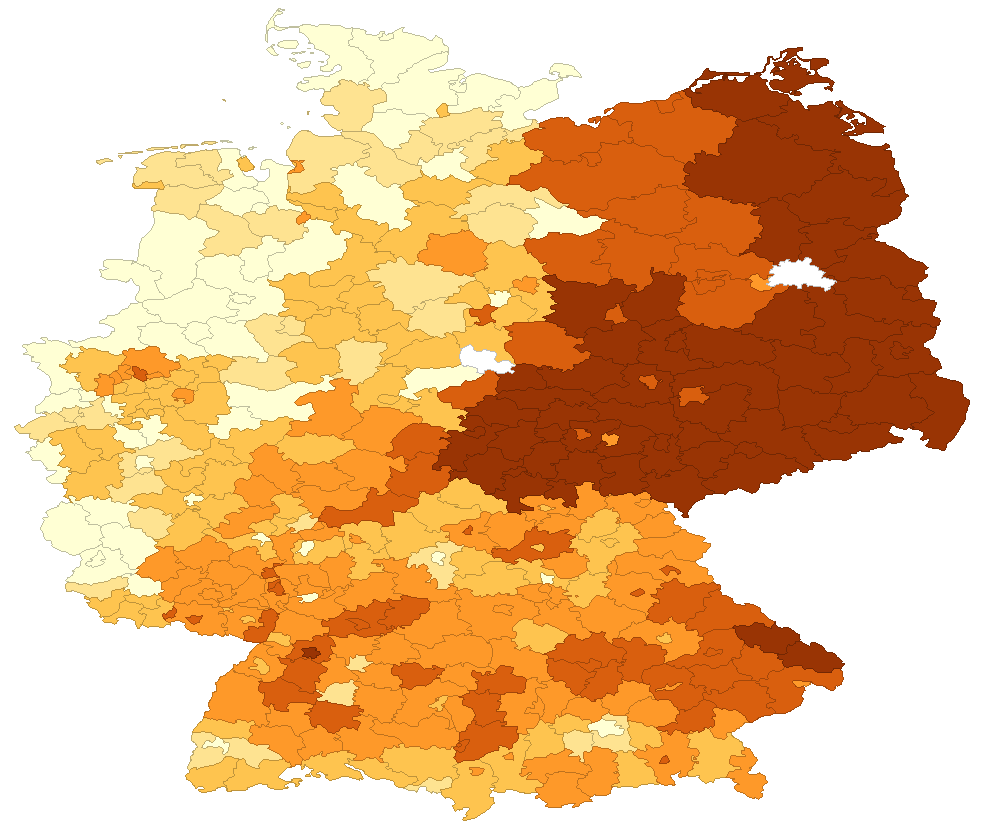 | | | | | | d 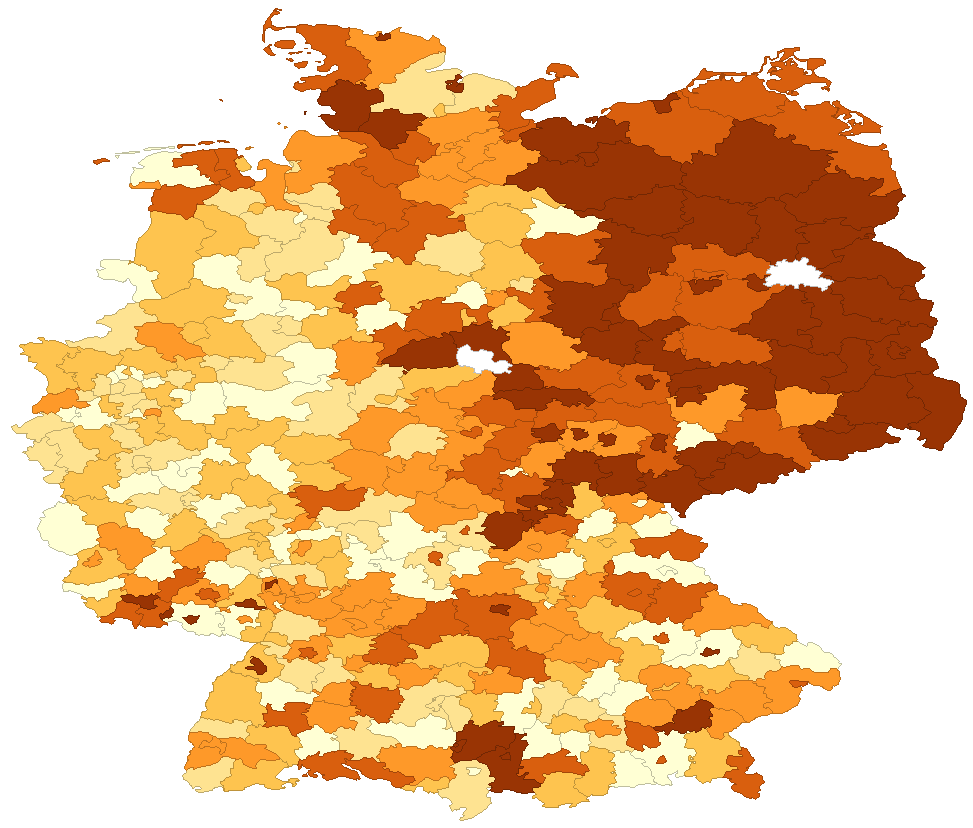 | | | | | |
| < .08 | .09 | [.10;.11] | [.12;.14] | [.15;.18] | > .19 | < .03 | [.04;.09] | [.10;.17] | [.18;.27] | [.28;.50] | > .51 |

Path model of socio-structural correlates of far right electoral support and political hate crimes in Germany (square-root transformed data).

Proportion of Foreigners

AfD Electoral Support

Right-wing Crime

Unemployment Rate

.04

.29

.20

-.33

-.46

.33

*R*^2^ = .26

*R*^2^ = .20

*Note*. Standardized path coefficients; error terms are not displayed for the sake of clarity.

Solid lines represent significant paths and correlations, *p* < .001.

Path model of socio-structural and psychological correlates of right-wing electoral support and political hate crimes in Germany (square-root transformed data).

*R*^2^ = .24

AfD Electoral Support

Collective Deprivation

-.04

Unemployment Rate

.11*

Extreme Right-wing Attitudes

*R*^2^ = .21

.38

-.21

-.32

-.45

.28

.20

-.11*

.31

*R*^2^ = .20

Intergroup Contact

Right-wing Crime

.32

Proportion of Foreigners

.06

*Note*. Standardized path coefficients; error terms are not displayed for the sake of clarity.

* *p* < .05, all other solid lines represent significant paths and correlations, *p* < .01.

Ordinary least squares regression results for socio-structural correlates of far right electoral support (top panel) and political hate crimes in Germany (bottom panel).

|  | AfD Electoral Support | | | | | | |
| --- | --- | --- | --- | --- | --- | --- | --- |
|  | Coeff. | S.E. | *t* |  | Coeff. | S.E. | *t* |
| Constant | .14 | .01 | 18.69** |  | .14 | .02 | 7.95** |
| Unemployment rate | .52 | .10 | 5.45** |  | .48 | .10 | 5.00** |
| Proportion of foreigners | -.41 | .05 | 8.35** |  | -.37 | .05 | 7.36** |
| Collective Deprivation |  |  |  |  | -.01 | .004 | 1.63 |
| Contact |  |  |  |  | -.01 | .004 | 1.61 |
| Extreme Right-wing Attitudes |  |  |  |  | .01 | .004 | 3.16* |
| *R*^2^ | .22 | | |  | .26 | | |
| AIC | -1130.71 | | |  | -1139.76 | | |
|  | Right-wing Crime | | | | | | |
|  | Coeff. | S.E. | *t* |  | Coeff. | S.E. | *t* |
| Constant | .25 | .06 | 4.47** |  | .14 | .13 | 1.04 |
| Unemployment rate | 4.88 | .71 | 6.88** |  | 4.86 | .72 | 6.75** |
| Proportion of foreigners | -2.41 | .36 | 6.64** |  | -2.33 | .38 | 6.13** |
| Collective Deprivation |  |  |  |  | .01 | .03 | < 1 |
| Contact |  |  |  |  | -.004 | .03 | < 1 |
| Extreme Right-wing Attitudes |  |  |  |  | .04 | .03 | 1.24 |
| *R*^2^ | .21 | | |  | .22 | | |
| AIC | 227.28 | | |  | 230.79 | | |

* *p* < .05, ** *p* < .01

Geographically weighted regression results for socio-structural correlates of far right electoral support (top panel) and political hate crimes in Germany (bottom panel) in East and West German municipalities separately.

|  | AfD Electoral Support | | | | | | |
| --- | --- | --- | --- | --- | --- | --- | --- |
|  | East | | |  | West | | |
|  | b | S.E. | *z* |  | b | S.E. | *z* |
| Constant | .23 | .02 | 10.82** |  | .10 | .01 | 13.71** |
| Lambda | .83 | .06 | 13.74** |  | .85 | .03 | 27.43** |
| Unemployment rate | .44 | .17 | 2.65** |  | .36 | .07 | 5.01** |
| Proportion of foreigners | -1.19 | .15 | 7.91** |  | -.10 | .03 | 2.76** |
|  |  | | |  |  | | |
|  | Right-wing Crime | | | | | | |
|  | East | | |  | West | | |
|  | b | S.E. | *z* |  | b | S.E. | *z* |
| Constant | -.22 | .26 | < 1 |  | .12 | .04 | 3.26** |
| Lambda | .42 | .13 | 3.26** |  | .41 | .07 | 6.13** |
| Unemployment rate | 8.20 | 3.11 | 2.64** |  | 1.02 | .60 | 1.70^†^ |
| Proportion of foreigners | 8.48 | 3.05 | 2.77** |  | .13 | .30 | < 1 |

^†^ *p* = .09, ** *p* < .01
